# Decoding mitochondrial DNA damage and repair associated with *H. pylori* infection

**DOI:** 10.3389/fcimb.2024.1529441

**Published:** 2025-01-21

**Authors:** Aashirwad Shahi, Dawit Kidane

**Affiliations:** Department of Physiology and Biophysics, College of Medicine, Howard University, Washington, DC, United States

**Keywords:** mitochondrial DNA damage and repair, *H. pylori*, genomic instability, cytosolic DNA, innate immune signaling, Type I interferon response, base excision DNA repair, cGAS-STING

## Abstract

Mitochondrial genomic stability is critical to prevent various human inflammatory diseases. Bacterial infection significantly increases oxidative stress, driving mitochondrial genomic instability and initiating inflammatory human disease. Oxidative DNA base damage is predominantly repaired by base excision repair (BER) in the nucleus (nBER) as well as in the mitochondria (mtBER). In this review, we summarize the molecular mechanisms of spontaneous and *H. pylori* infection-associated oxidative mtDNA damage, mtDNA replication stress, and its impact on innate immune signaling. Additionally, we discuss how mutations located on mitochondria targeting sequence (MTS) of BER genes may contribute to mtDNA genome instability and innate immune signaling activation. Overall, the review summarizes evidence to understand the dynamics of mitochondria genome and the impact of mtBER in innate immune response during *H. pylori*-associated pathological outcomes.

## Introduction

Mitochondria are essential organelles responsible for energy production and maintaining calcium homeostasis, lipid, and amino acid metabolism (Casanova et al., 2023). The human mitochondria DNA (mtDNA) is present in multiple copies per cell (Filograna et al., 2021). Targeting mitochondria has emerged as a key strategy for bacteria to hijack host cell physiology and promote infection (Blanke, 2005; Fielden et al., 2017). Numerous pathogenic bacteria have evolved strategies to subvert the mitochondrial functions of host cells to support their own proliferation and dissemination (Galmiche et al., 2000; Fischer et al., 2004; Stavru et al., 2011). In addition, bacteria can modulate mitochondrial functions to access nutrients and/or evade the host’s immune system (Spier et al., 2019). Infection by extracellular pathogens including *H. pylori* is able to change the mitochondrial metabolic and oxidative profile of infected cells (Andrieux et al., 2021). Furthermore, a study has shown that *H. pylori* infection induces genetic dysfunction in both nDNA and mtDNA (Hiyama et al., 2003).

Notably, mtDNA is a hotspot for constant insult from both exogenous and endogenous stresses (Alexeyev et al., 2013). Cellular and biochemical evidence suggests that mtDNA is more susceptible to oxidized DNA damages than nuclear DNA due to its proximity to the sites of oxidative phosphorylation and lack of protection by histones (Yakes and Van Houten, 1997; Druzhyna et al., 2008). Excessive accumulation of mtDNA damages leads to mitochondrial dysfunction and provokes the pathogenesis of many human diseases, including neurodegeneration, cancer, and diabetes (Wallace, 2005; Nakabeppu et al., 2007; Llanos-Gonzalez et al., 2019). Oxidative DNA damage lesions in mtDNA and/or mtDNA replication blocks are removed by different types of DNA damage repair enzymes (LeDoux et al., 1992; Zhao and Sumberaz, 2020). Most of the repair proteins and/or enzymes are imported from the nucleus, where they process oxidative mtDNA lesions and promote repair (Bohr, 2002; de Souza-Pinto et al., 2009; Gredilla, 2010). However, the loss of these nuclear and mitochondria-encoded repair proteins significantly impairs repair efficiency in mitochondria (Lia et al., 2018). Therefore, the role and function of mitochondrial oxidative DNA damage repair are not expected to be independent of nuclear BER.

In eukaryotic cells, mtDNA molecules are organized into several hundred nucleoids (Legros et al., 2004; Wang and Bogenhagen, 2006; Bogenhagen, 2012; Prachar, 2016), which function as units of mtDNA propagation for replication, segregation, and gene expression (Spelbrink, 2010; Ban-Ishihara et al., 2013; Kolesnikov, 2016). Several proteins are involved in maintaining the integrity of mitochondrial genome replication, including DNA polymerase γ (POLG), TWINKLE (DNA helicase), mitochondrial RNA polymerase (POLRMT), mitochondrial single-stranded DNA-binding protein (mtSSB), RNASEH1, DNA ligase III, mitochondrial genome maintenance exonuclease1 (MGME1), flap endonuclease 1 (FEN1), and topoisomerase (Sharma and Sampath, 2019; Fontana and Gahlon, 2020). POLG plays a significant role in maintaining mtDNA replication integrity and participates in base excision repair. Moreover, POLG has 3′–5′ exonuclease and 5′-deoxyribose phosphate (dRP) activities associated with its catalytic subunit (Kaguni, 2004; Graziewicz et al., 2006). POLG’s polymerase activity is critical to synthesize DNA, and it also has a weak dRP lyase function that is complemented by DNA polymerase beta (POLB) dRP lyase activity (Longley et al., 1998; Sykora et al., 2017). Furthermore, the primase activity of PrimPol initiates *de novo* DNA synthesis using deoxynucleotide while discriminating against ribonucleotides (Martinez-Jimenez et al., 2018; Diaz-Talavera et al., 2022). Other DNA repair factors, such as mitochondrial single-stranded binding protein 1 (SSBP1), protect the active replicative DNA regions (Guilliam et al., 2015). Based on several studies, three different models have been proposed for mtDNA replication (Robberson et al., 1972; McKinney and Oliveira, 2013). Among these three models, the strand-displacement model (SDM) is the most accepted model because it best explains the dynamics of mtDNA replication. According to this model, replication starts at the oriH site and proceeds unidirectionally until it reaches the origin of light strand (oriL). At this point, the synthesis of light strand begins in the opposite direction, continuing until the replication of both strands is complete. Importantly, mutations in the mitochondrial replisome’s proteins POLG, TFAM, and MGME1 genes are associated with the accumulation of mtDNA deletions that may also increase susceptibility for infection-induced chronic-inflammation-associated disease (Spelbrink et al., 2001; Longley et al., 2006; Nicholls et al., 2014; Fontana and Gahlon, 2020). In the next section of this manuscript, we will address key questions such as (i) how do host cells handle oxidative stress-associated mtDNA damage via BER in the presence and absence of bacterial infection, (ii) how do oxidative-stress-induced base lesions or repair intermediates impact mtDNA replication dynamics, and (iii) does infection by extracellular bacteria, such as *H. pylori*, induce mtDNA-mediated innate immune signaling?

## mtDNA damage and BER in mitochondria

Upon bacterial infection, a major challenge for host cells is the maintenance of genomic integrity. Pathogenic bacteria can cause DNA damage in host cells, often resulting in DNA double-strand breaks (DSBs) (Cancer Genome Atlas Research N, 2014; Song and Bent, 2014). Numerous studies have reported that *H*. *pylori* infection induces DNA damage and alter the DNA repair capacity (Dorer et al., 2010; Lieber, 2010; Toller et al., 2011; Chaturvedi et al., 2014; Koeppel et al., 2015). *H*. *pylori* has been found to cause several types of DNA damage, including single-strand breaks (SSBs) and DSBs in nuclear genome (Fox and Wang, 2007; Lieber, 2010). High-throughput genomic analyses have shown that *H*. *pylori* causes a specific pattern of DNA damage in the transcribed and telomere-proximal regions of the genome (Chaturvedi et al., 2014). Furthermore, *H. pylori* infection induces mtDNA damage that includes oxidative damage, adducts formation, base mismatch, and DNA strand breaks (Babbar et al., 2020). Given its proximity to ROS-generating electron transport chain and the absence of histones, mtDNA is more vulnerable to oxidative DNA damage than nDNA (Maynard et al., 2009). Oxidative damage to mtDNA can manifest as base modifications, abasic sites, and various other types of lesions (Cooke et al., 2003). One of the most studied lesions in mtDNA is 8-oxoguanine (8-oxoG), which is a mutagenic lesion (Kurosaka et al., 1991). Mispairing of 8-oxoG with adenine results in a G–C to T–A transversion during subsequent rounds of replication. Early studies showed that 8-oxoG lesions are 16 times more frequent in mtDNA than in nDNA (Richter et al., 1988). In more definitive studies, Yakes and Van Houten showed that mtDNA damage is more extensive and persists longer than nDNA damage in human cells following oxidative stress (Yakes and Van Houten, 1997). In addition, unrepaired mtDNA base damage intermediates, such as single-stranded strand breaks (SSBs), arise as a result of the erroneous or abortive activity of DNA topoisomerase I (Hudson et al., 2012), contributing to mitochondrial genome instability (Zhang et al., 2001). In addition, *H. pylori* infection may also lead to replication stress in mtDNA that may eventually alter the expression and function of mitochondrial genes and transcription factors that contribute to the accumulation of mtDNA damage (Chatre et al., 2017). It is also possible that the enhanced oxidative stress due to *H. pylori* infection might be a possible cause of unfit mitochondria for replication in infected host cells. Another important factor for increased mitochondrial DNA damage is mtDNA mutations that occur during replication by insertion/deletion of the wrong nucleotide. Although the POLG has 3′–5′ exonuclease proofreading activity that corrects the mis-incorporation of the nucleotide, the error rate of mDNA replication, however, exceeds the repair capacity, potentially increasing the mutation frequency (Kaguni, 2004). Moreover, *H. pylori* induces genomic instability in nuclear CA repeats in mice and in mtDNA (MaChado et al., 2009).

**Figure 1:**
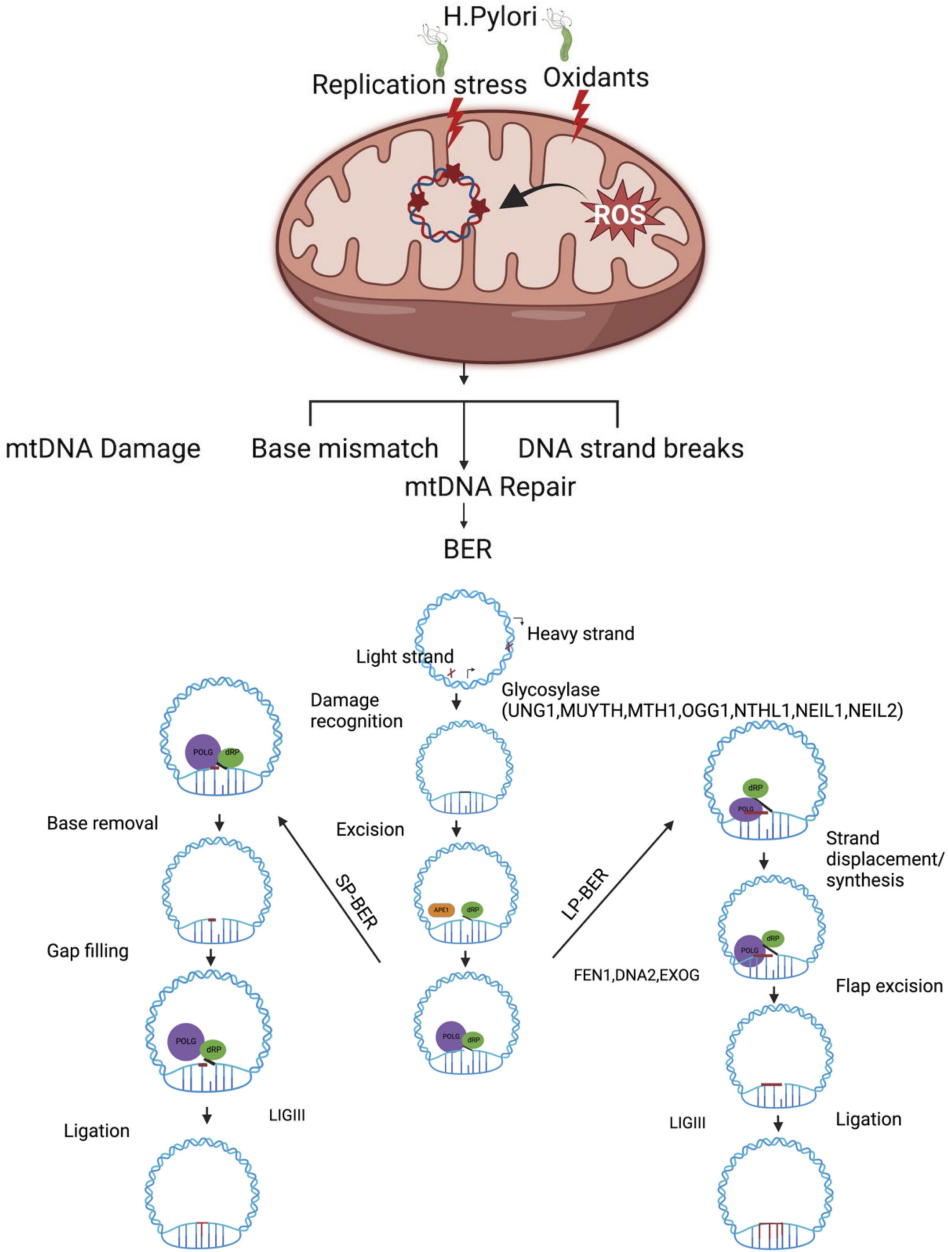
Oxidative stress induced by *H. pylori* infection leads to damage in mitochondrial DNA (mtDNA), which is primarily repaired through the base excision repair (BER) pathway. The BER pathway operates through two different mechanisms to maintain the mitochondrial genome: short-patch BER (SP-BER) removes a single damaged nucleotide, while long-patch BER (LP-BER) removes between two and eight damaged nucleotides during the repair process. *H. pylori* infection induces genotoxin-mediated mtDNA and increases oxidative-stress-associated mtDNA damage and mtDNA replication stress. A single-base damage or single-strand break on mtDNA is likely processed via BER. mtDNA single-base damage is potentially recognized and removed by one of the DNA glycosylases (UNG1, OGG1, MUTHY MTH1, NTHL1 NEIL1, and NEIL2), followed by end processing via the dRP lyase activity of POLB and gap filling with POLG inserts in the correct base, and LIGII seals the mtDNA nick. In long-patch BER, strand displacement DNA synthesis is processed by POLG and displaces 5′ DNA flap downstream of the repair site, which must be removed by flap endonuclease (FEN1) and other partner/DNA2/EXOG involved to process the 5′ end of the DNA. Once tailoring of the 5′ and 3′ ends of mtDNA is complete, LIGIII seals the mtDNA nick. Figure created with BioRender.com.

Although various DNA repair pathways have been documented including direct reversal, BER, NER, and MMR in cells (Jalal et al., 2011; Chatterjee and Walker, 2017), the BER pathway is the predominant pathway for repairing mtDNA damage (Bohr and Anson, 1999; Druzhyna et al., 2008). Like nDNA, an efficient mtDNA repair pathway, especially the BER pathway, may play an important role in repairing oxidative mtDNA damage (**Figure 1**). Mitochondria BER (mtBER) proteins are localized in the inner membrane and co-exist with the TFAM nucleoid structure protein (Stuart et al., 2005). The first step of mtBER involves DNA base damage recognition by seven different DNA glycosylases. These glycosylases contain a mitochondria translocation signaling (MTS) leader sequence, which facilitates their transport into the mitochondria. Once inside, these DNA glycosylases remove damaged mtDNA nucleotide lesions. The second step involves cleaving the sugar–phosphate backbone of the mtDNA using AP endonuclease that processes the abasic site (AP). This is followed by the action of POLG, which re-synthesizes missing DNA patches. Finally, DNA ligase (LIG3) seals the DNA fragments (Szczepanowska and Trifunovic, 2015). The alternative mechanism is that mtDNA repair machinery engages in end processing using distinct gap-tailoring enzymes, including aprataxin (Ahel et al., 2006) and TDP1 (Das et al., 2010). However, if aprataxin proteins are unable to repair the 5′-AMP group, it can block DNA ligase repair activity and generate SSBs (Sykora et al., 2011). The mtDNA damage induced by *H. pylori* infection may lead to mtDNA single-strand breaks (mtSSBs), mtDNA double-strand breaks (mtDSBs), and base mismatches which are potentially processed via different types of repair machinery (**Figure 1**). Due to the types of oxidative DNA damage substrate specificity, the preference of DNA glycosylase may vary, and it is possible that they might influence each other’s activity (MaChado et al., 2009). The DNA glycosylases OGG1, UDG1, and MYH (Ohtsubo et al., 2000) are all associated with the particulate fraction of the mitochondria as are POLG, DNA ligase III, and a minor portion of AP endonuclease activity (Stuart et al., 2005). The mitochondria harbor bifunctional 8-oxoguanine, DNA glycosylase-1 (OGG1), and monofunctional uracil–DNA glycosylase (UNG1) to process different mtDNA base lesions (Jacobs and Schar, 2012). These glycosylases are discussed below.

## DNA glycosylase in mitochondria

Several studies have identified five bifunctional and two monofunctional DNA glycosylases in the mitochondria (Prakash and Doublie, 2015). Uracil–DNA glycosylase 1 (UDG1 or uracil-*N*-glycosylase1 [UNG1]) (Anderson and Friedberg, 1980) and MUTYH (MYH), a homolog of the *Escherichia coli* MutY glycosylase (Ohtsubo et al., 2000), are classified as monofunctional DNA glycosylases. The substrate specificities of UNG1 and MUTYH have been recently reviewed (Svilar et al., 2011). MUTYH is an adenine–DNA glycosylase that preferentially excise adenine when paired with 8-oxoG, initiating a round of base excision repair that restores the 8-oxoG:C pair and protects the DNA from mutagenic 8-oxoG lesions (Michaels et al., 1992). In addition, several studies have shown that mitochondria can repair alkylation lesions using monofunctional glycosylase, MPG (Chakravarti et al., 1991; Pirsel and Bohr, 1993; Ledoux et al., 1998). The UNG1 enzymes cleave substrates from both single-stranded (ss) DNA and double-stranded (ds) DNA with a slight preference for ss over ds substrates. Importantly, UNG1 has a MTS comprising a 30-amino-acid leader sequence at the N-terminal end of the enzyme that likely facilitates entry into the inner mitochondrial membrane (Neupert, 1997). Amino acid substitution (Y147A or N204D) in the catalytic domain of UNG1 switches the substrate specificity of the enzyme and is able to remove thymine and uracil from mtDNA (Kavli et al., 1996). Removing mtDNA base lesions in this manner leaves excess apyrimidinic sites, which are highly genotoxic to the cells (Glassner et al., 1998; Lindahl and Wood, 1999). mtDNA has been shown to accumulate high levels of mutagenic lesions of 8-hydroxy-2′-deoxyguanosine, which is the byproduct of guanine hydroxylation (Nakabeppu, 2014). Previous work has shown that 8-oxodG, the most prominent oxidative DNA base lesion, is repaired more efficiently in the mitochondria than in the nucleus (Thorslund et al., 2002). These 8oxoG lesions are recognized and processed by OGG1 glycosylase (Mandal et al., 2012) which localizes to both the nucleus and mitochondria (Klungland et al., 1999; Nishioka et al., 1999; Klungland and Bjelland, 2007). However, the loss of OGG1 compromises the metabolic function of mitochondria, indicating an additional role in maintaining the bioenergetic homeostasis of the cell (Lia et al., 2018). Notably, other DNA glycosylases such as NTHL1 are found in both the nucleus and mitochondria and only active with duplex DNA. NTHL1 is a bifunctional glycosylase involved in the excision of oxidized DNA bases such as Tg, 5-hydroxycytosine (5-hC), 5-hydroxyuracil (5-hU), and the ring-opened 2,6-diamino-4-hydroxy-5-formamidopyrimidine (Fapy) lesions (Prakash and Doublie, 2015). Previously, we have shown that the single-nucleotide variant of NTHL1 promotes genomic instability in cells (Galick et al., 2013). However, the biological significance of this mutant variant in mitochondria is unclear and requires further investigation. Additionally, chromatin immunoprecipitation analysis demonstrated that DNA glycosylases, including *NEIL1* and *NEIL2*, form a complex with mitochondrial genes MT-CO2 and MT-CO3 (cytochrome c oxidase subunit 2 and 3) and mitochondrion-specific POLG (Mandal et al., 2012). NEIL2 interacts with PNK to maintain the mammalian mitochondrial genome (Mandal et al., 2012). NEIL2 shows a unique preference for excising lesions from a DNA bubble. In contrast, NEIL1 efficiently excises 5-hydroxyuracil, an oxidation product of cytosine, from the bubble and single-stranded DNA but does not have strong activity toward 8-oxoguanine in the bubble (Dou et al., 2003). Furthermore, MTH1 DNA glycosylase, which is localized in both the mitochondria and nucleus, plays a significant role in repairing oxidized dATP and ATP, such as 2-OH-dATP and 2-OH-ATP, as well as 8-oxo-dGTP (Bialkowski and Kasprzak, 1998; Fujikawa et al., 1999; Fujikawa et al., 2001; Nakabeppu et al., 2006). The function of those nuclear-encoded DNA glycosylases likely depends on their ability to pass through the mitochondrial membrane via MTS signals. However, there are single-nucleotide polymorphisms (SNPs) on the MTS of these glycosylases that may impact their function and cause mitochondrion-associated human diseases (**Table 1**). Uncovering the biological significance of these SNPs will likely shed mechanistic insights on the impact of DNA glycosylase in mitochondrial genome integrity and its biological outcomes.

**Table 1:** Variants associated with mutation on mitochondrial targeting sequence (MTS) of base excision repair (BER) genes and its clinical significance.

Gene	MTS location	Position changed	Variation	Variant id	Mutation description	Clinical significance	References
OGG1	8-21	9	p.Arg9Ser	rs769947581	Missense, Benign (uniprot)	Unknown	PMID:29848661
		12	p.Gly12Glu	rs772520254	Missense, Benign (Uniprot)	Unknown	MTSviewer
MTH1	1-18	2	pGly2Asp	rs144573336	Missense (Uniprot)	Unknown	PMID: 16607562
		17	pArg17Gln	rs372407158	Somatic, Missense (Uniprot)	Unknown	MTSviewer
UNG	1-35	11	p.Phe11Ser	947219	Germline, Missense (ClinVar)	Hyper IgM syndrome type 5	PMID: 9776759
		21	p.Ala21Thr	643750	Germline, Missense (ClinVar)	Hyper IgM syndrome type 5	MTSviewer
MUYTH	1-14	1	p.Met1Val	230848	Germline, Missense, Pathogenic (ClinVar)	Familial adenomatous polyposis 2/ Hereditary cancer- predisposing syndrome/Gastric cancer Familial adenomatous polyposis 2.	PMID:21235684
		12	p.Trp12Ter	483936	Germline, Nonsense, pathogenic (ClinVar)	Familial adenomatous polyposis 2	MTSviewer
NTHL1	1-95	18	p.Thr10Ser	657414	Germline, Missense, likely Benign (ClinVar)	Familial adenomatous polyposis 3/ Hereditary cancer- predisposing syndrome	PMID:9611236
		62	p.Gln54Ter	662775	Germline, Missense, pathogenic (ClinVar)	Familial adenomatous polyposis 3/ Hereditary cancer- predisposing syndrome	MTSviewer
NEIL1	1-89	68	p.Pro68His	rs187873972	Missense (Uniprot)	Unknown	PMID:2575473
		24	p.Gly24Cys	rs761525934	Missense (Uniport)	Unknown	MTSviewer
NEIL2	No canonical MTS	N/A	N/A	N/A	N/A	N/A	PMID:22130663, PMID: 25754732 MTSviewer
APEX1	289-318	291	p.L291Vfs*6	rs747329195	Somatic, Frameshift (Uniprot)	Unknown	PMID:20231292
		307	p.Ser307Asn	rs1183577581	Missense (Uniport)	Unknown	MTSviewer
POLG	1-25	10	p.Ala10Val	458708	Germline, Missense, Benign (ClinVar)	Progressive sclerosing poliodystrophy	PMID 8884268 PMID: 18546365
		11	p.Gly11Ser	619334	Germline, Missense, Benign (ClinVar)	Progressive sclerosing poliodystrophy	MTSviewer
POLB	1-17	8	p.Gln8Arg	Rs200636493	Missense, Benign (Uniprot)	Unknown	PMID:28559431
		7	p.Pro7ser	Rs1463614564	Missense, Benign (Uniprot)	Unknown	MTSviewer
LIG3	73-333	224	p.Arg224Trp	782153	Germline, Missense, Benign (ClinVar)	Unknown	PMID:10207110
		241	p.Ser241Leu	987864	Germline, Missense, Benign (ClinVar)	Unknown	MTSviewer

This table summarizes the mutations within the mitochondrial targeting sequence (MTS) of various base excision repair (BER) genes, along with the positions of the amino acid changes, corresponding variant IDs, and their clinical significance based on databases ClinVar and UniPort as well as software MTSviewer and existing published literature.

## APE1 endonuclease

APE1 is a multifunctional protein that plays a central role in the maintenance of nuclear and mitochondrial genomes. APE1 translocates into the mitochondria in response to oxidative stress and increases mitochondrial DNA (mtDNA) repair rate and cell survival (Barchiesi et al., 2020). Protein sequence analysis suggests that APE1 harbors MTS signal sequence within residues 289–318 in the C terminus, which is normally masked by the intact N-terminal structure (Li et al., 2010). Once APE1 is translocated in the mitochondria, it is able to remove the AP sites and hand over the reaction to the next repair factors. In contrast, genetic ablation of APE1 results in the accumulation of damaged mitochondrial mRNA species, impairment in protein translation, and reduced expression of mitochondrial encoded proteins, leading to less efficient mitochondrial respiration (Barchiesi et al., 2020). It is possible that loss of APE1 may increase the number of AP sites, potentially driving mtDNA instability. A few studies suggested that APE1 depletion in cells leads to increased mtDNA copy number (Barchiesi et al., 2021).

## DNA polymerase enzymes

The ability to effectively repair various types of DNA damage is achieved through multiple, often overlapping, DNA repair pathways. DNA POLB and POLG are involved in mtDNA repair process (Copeland, 2010). Once the AP site is processed by APE1, the gap is filled by POLG with correct nucleotides. The Wilson study estimated that ~30% of POLB localize to the mitochondria, as shown through the colocalization studies of TOM20 (Prasad et al., 2017). Additional high-quality immunogold electron microscopy (EM) localization studies demonstrated that 20% of POLB localize to the mitochondrial matrix and 60% to the nucleus (Prasad et al., 2017). POLG has DNA polymerase activity to fill DNA gaps but lacks efficient dRP lyase activity to process the 5′dRP groups (Kaufman and Van Houten, 2017). Bohr’s and Wilson’s groups identified a robust dRP lyase activity in the mitochondria belonging to POLB (Sykora et al., 2017). Biochemical characterization indicates that the 5′dRP lyase activity of DNA polymerase beta plays a primary role in complementing POLG by removing the 5′dRP group, thus promoting short-patch-BER in mtDNA. Both POLB and POLG support gap filling in single nucleotide gaps (Kaufman and Van Houten, 2017). POLG is known for its high replication fidelity, which allows it to support both replication and repair functions in the mitochondria. This high fidelity, however, may be detrimental in situations that require the polymerase to bypass a lesion.

## DNA ligase

DNA LIG III is a key factor of the BER pathway which is shared between the mitochondria and the nucleus compartment, where it is involved in sealing DNA nicks to complete mtDNA repair processes. LIG3 is the only vertebral mitochondrial DNA ligase identified so far and is essential for mitochondrial DNA maintenance (Gao et al., 2011; Simsek et al., 2011). In the mitochondria, LIG3 interacts with tyrosyl-DNA phosphodiesterase 1 (TDP1), NEIL1/2 glycosylases, and POLG (Simsek and Jasin, 2011). *In vitro* work shows that downregulation of LIG3 in human fibroblastoma cell lines decreased the mtDNA copy number, reduces respiration, and leads to the accumulation of DNA SSBs in mtDNA. In contrast, the complete lack of LIG3 in murine cells leads to the full depletion of mtDNA, underlying the essential role of LIG3 in mitochondrial genome integrity (Lakshmipathy and Campbell, 2001; Shokolenko et al., 2013). The somatic and germline variants of LIG3 may contribute to the loss of function and accumulation of mtDNA damage which likely drives mitochondrion-associated human pathologies.

## Impact of aberrant BER repair on mitochondrial genomic integrity

Loss of BER results in the accumulation of mutation [(C:G→T transversions] (Whitaker et al., 2017) or DNA single-strand (Lindahl, 1993) or double-strand breaks (DSBs)] (Woodbine et al., 2011; Fridlich et al., 2015), which are principal sources of genomic instability (Khanna and Jackson, 2001; Caldecott, 2008). Dysfunctional mtBER leads to the accumulation of mtDNA D-loop mutation in gastrointestinal cancer (Wang et al., 2018). DNA-repair-deficient mitochondria are more susceptible to oxidative DNA damage agents (Shokolenko et al., 2003). It is possible that loss or mutation in MTS signaling sequence contributes to the lack of mtBER in the mitochondrial compartment. Mutations in MTS of BER genes may prevent the import of the nuclear encoded BER proteins into the mitochondria, resulting in the loss of their biological functions in the mitochondria. Germline and somatic variants of BER genes that harbor MTS mutations likely cause deficiency in mtBER repair pathways, contributing to mitochondrial genome instability and human diseases (**Table 1**). Germline BER variants with non-synonymous mutations in the MTS sequence likely increase the risk factor for different pathophysiological outcomes. Similarly, mutations in BER genes within tumors may contribute to tumor initiation and progression. It is important to note that the genetic mutations in MTS, analyzed using the MTSViewer platform, suggested MTS mutation sites, and clinical variant scores likely suggest the potential impact of these mutations on protein structure and function in the mitochondria.

## Impact of *H. pylori* infection on mitochondrial genome transactions

**Figure 2:**
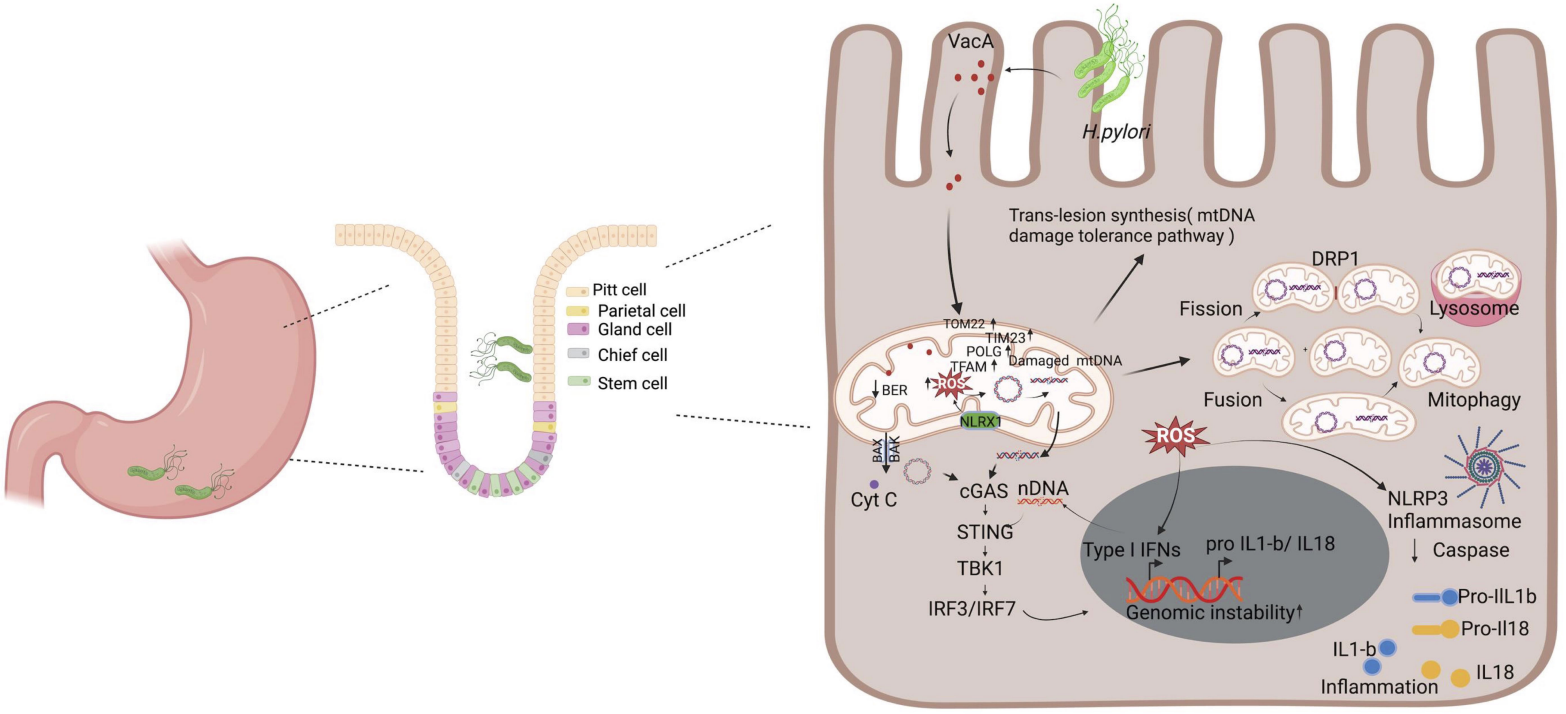
*H. pylori*-mediated mitochondrial dysfunction and inflammation. Upon infection, *H. pylori* secretes toxins such as VacA, which interacts with the mitochondria, leading to the modulation of its function and ultimately promoting pathogenesis. It decreases the mitochondrial membrane potential, leading to reduced ATP production and an increase in cytochrome c release that triggers autophagy. Additionally, VacA enhances the mtDNA damage and the generation of ROS. This triggers a series of stress responses, including the upregulation of mitochondrial DNA repair mechanism factors (e.g., POLG and TFAM) and the activation of the cGAS/STING pathway due to the release of damaged mtDNA and nDNA in the cytosol. Cells have a mechanism to respond to unrepaired mtDNA damages that includes trans-lesion synthesis, fusion, fission, and mitophagy that degrades severely damaged mitochondria. The accumulation of ROS and the release of mitochondrial contents also activate the NLRP3 inflammasome, leading to the processing and release of pro-inflammatory cytokines IL-1β and IL-18. Collectively, these processes contribute to chronic inflammation and genomic instability, which are key factors in the pathogenesis of *H. pylori*-related diseases, including gastritis and gastric cancer. Figure created with BioRender.com.

*H. pylori* infection causes chronic gastric inflammation (Peek and Blaser, 2002), and patients with a previous history of *H. pylori* infection are at a higher risk to develop gastric cancers (Aoi et al., 2006). Furthermore infection with *H. pylori* suppresses stomach acidity and may result in a more permissive milieu for colonization with other bacteria (Dicksved et al., 2009). Mitochondrial dynamics play important roles in bacterial pathogenesis, with multiple mitochondrial functions mechanistically linked to their morphology, which is defined by ongoing events of fission and fusion of the outer and inner membranes (Cogliati et al., 2016). *H. pylori* infection dysregulates the delicate balance of mitochondrial fission and fusion networks (Scott and Youle, 2010). Mitochondrial fusion allows the mitochondria with normal mtDNA to compensate for defects in the mitochondria with damaged mtDNA (Nakada et al., 2001; Ono et al., 2001; Yang and Gao, 2018). These processes are governed by a complex molecular machinery and finely tuned by regulatory proteins (Tilokani et al., 2018). *H. pylori*-induced mtDNA damage may contribute to trigger this event via genomic instability such as mutations and deletions in mitochondrial DNA that yield a heteroplasmic mixture of wild-type and mutant mitochondrial genomes within one cell (Taylor and Turnbull, 2005). As shown in **Figure 2**, the mtDNA that harbor extensive damage likely removed from the cellular system via mitochondria fission process to minimize the carryover of undesirable genetic traits to next cell cycle. Furthermore, mitochondrial fission is needed to create not only new mitochondria, but also contributes to quality control by enabling the removal of damaged mitochondria and can facilitate apoptosis during high levels of cellular stress. Therefore, mitochondrial fission is an important element to eliminate infected cells and reduce cell-to-cell-spreading, thus modulating apoptosis and bacterial dissemination (Spier et al., 2019). In contrast mitochondria harboring different genetic lesions likely compensate for their defects by relying on the genetic content from other mitochondria through the fusion process. Damaged and undamaged mtDNAs yield a heteroplasmic mixture of normal and mutant mitochondrial genomes within the same cell (Wonnapinij et al., 2008; Aryaman et al., 2018). The mitochondria fusion scenario likely maintained if the mutation rate in the mitochondria remain below ~ 80% per cell, the mitochondria in heteroplasmic cells complement one another to compensate their defects (Yoneda et al., 1994; Nakada et al., 2001). Mitochondrial Fusion can rescue two mitochondria with mutations in different genes through cross-complementation to one another, and it can mitigate the effects of *H. pylori* infection induced DNA damage by the exchange of repair proteins and other factors with other mitochondria. It is also important that mitochondrial fusion can therefore maximize oxidative capacity in response to toxic stress and use alternative resource or repair factors to fix the damaged region of mtDNA.

## *H. pylori* toxin-induced mitochondria dysfunction

Mitochondria play a central role in the innate immune response. It is at the center of the inflammatory response in the case of a viral or bacterial infection or spontaneous cellular damage. Because of their structural similarity to their bacterial ancestor, extracellular mitochondria and their components may operate as a danger signal by means of their interaction with pattern recognition receptors (PRRs). PRRs are a group of receptors that can specifically detect molecular patterns found on the surfaces of pathogens, apoptotic cells and damaged senescent cells. In the case of an infection by a pathogenic agent, the microorganisms will be detected by PRR that recognize pathogen-associated molecular patterns (PAMPs), such as flagellins, lipopolysaccharide, mannose, nucleic acids and proteins and the danger-associated molecular motifs (DAMPs) molecules. In addition, the presence of the bacterial virulence factors such as type IV secretion system (T4SS), the bacterial protein CagA and the vacuolating cytotoxin (VacA) is associated with chronic inflammation and increased risk of gastric cancer development (Peek and Blaser, 2002). *H*. *pylori* strains are categorized into *cagA*‐positive and *cagA*‐negative strains based on the presence or absence of the *cag* pathogenicity island (*cag*PAI). The *cag*PAI, is an ~40‐kb DNA segment containing around 30 genes (open reading frames), which include *cagA* and several genes encoding components of a bacterial Type IV secretion system (T4SS), that delivers CagA into attached gastric epithelial cells (Covacci and Rappuoli, 2000). Cag A is capable to induce cytosolic Ca^2+^ influx, leading to mitochondria ROS production. In addition, Cag A can upregulate the expression level of spermine oxidase (SMO), which can convert spermine to spermidine and simultaneously releases hydrogen peroxide (Chaturvedi et al., 2011; Cindrilla et al., 2016).

*H*. *pylori* is known to target mitochondria through its vacuolating cytotoxin (VacA), which triggers mitochondria-dependent apoptosis in mammalian cells (Calore et al., 2010). In gastric epithelial cells, VacA localizes to endosomal compartments and reaches the mitochondrial inner membrane where it forms anion-conductive channels (Calore et al., 2010; Domanska et al., 2010). VacA reduces mitochondrial membrane potential leading to decreased ATP production and cytochrome c release (Galmiche and Rassow, 2010). The pore-forming VacA toxin of the *H. pylori*, recruits and activates Drp1 resulting in mitochondrial fission, Bax activation, MOMP and cytochrome *c* release (Jain et al., 2011). VacA is also an efficient inducer of autophagy (Terebiznik et al., 2009). It is possible that *H*. *pylori* deregulate host cell mitochondria at early and late stage of infection with different dynamics. At the early stage of infection, *H. pylori* induce VacA dependent dysregulation of mitochondria hemostasis, which promotes transient increase in mitochondrial translocases, mitochondrial DNA replication maintenance factors such as POLG and TFAM. In contrast, at late infection stage the mechanism of dysregulation is VacA independent alteration in mitochondrial replication and import components, suggesting the involvement of additional H. *pylori* activities in mitochondrion-mediated effects (**Figure 2**).

## mtDNA modulates *H. pylori* infection-associated inflammation

Mitochondria have been reported as modulators of cellular antibacterial immunity and inflammatory response (Andrieux et al., 2021). Abundant lines of research implicate the mitochondria as a key immune modulator in mouse models and human materials. Components of mtDNA such as TFAM, extracellular ATP, and numerous others have the capacity to elicit strong immune responses and, as such, and are thus considered mitochondrial damage-associated molecular patterns (DAMPs) (Galluzzi et al., 2012; West et al., 2015; De Gaetano et al., 2021). Mitochondrial DNA (mtDNA) encodes essential subunits of the oxidative phosphorylation system and is also a major damage-associated molecular pattern (DAMP) that engages innate immune sensors when released into the cytoplasm, outside of cells or into the circulation. As a DAMP, mtDNA not only contributes to anti-viral resistance but also causes pathogenic inflammation in many disease contexts. Several studies also report that when mtDNA is discharged outside the cell, whether intact or damaged, it shows considerable pro- or anti-inflammatory effects in different models, thus highlighting the paradoxical interactions between these organelles and immune cells (Boudreau et al., 2014; Torralba et al., 2016). Mitochondrial DNA released into the cytosol is recognized by a DNA sensor cGAS, a cGAMP/STING which activates a pathway leading to the enhanced expression of type I interferons (**Figure 2**). Additionally, mtDNA activates NLRP3 inflammasome, which promotes the activation of pro-inflammatory cytokines interleukin-1 beta and interleukin-18 (West et al., 2015; Zhong et al., 2018; Swanson et al., 2019). In the endosome, mtDNA can also bind to Toll-like receptor-9, triggering a pathway that results in the expression of pro-inflammatory cytokines (De Gaetano et al., 2021). Stress-induced release of mtDNA or mtRNA into the cytoplasm can activate a type I IFN-I response that confers resistance to viral infection (West et al., 2015; Dhir et al., 2018; Sprenger et al., 2021). Inflammation caused by infection leads to the production of ROS and subsequent oxidative DNA damage (Sahan et al., 2018). ROS partially derives from active immune systems and host cells (Cindrilla et al., 2016). During infection, the stimulation of phagocytic cells, such as neutrophils, eosinophils, monocytes, and macrophages, activates the NADPH oxidase (Nox) pathway, which catalyzes the reduction of oxygen using NADPH and generates superoxide (Brown and Griendling, 2009). In infected cells, the production of ROS is further amplified in the mitochondria via a mechanism involving NLRX1, a member of the intracellular Nod-like receptor (NLR) family that is localized in the mitochondria (Abdul-Sater et al., 2010). The resulting ROS can enter the nucleus and attack the DNA, generating oxidative DNA damage, such as 8-oxo-G, AP sites, and single-strand breaks (SSBs) (Kidane et al., 2014). Overall, further work is needed to uncover whether mtDNA and/or nuclear DNA damage continuously provides the fuel to exacerbate *H. pylori* infection-mediated inflammation.

## Future perspective

Mitochondrial DNA integrity is critical to keep cellular homeostasis and prevent undesirable immune activation. Spontaneous or exogenous-stress-mediated mtDNA damage triggers different types of mitochondrial responses including fission or fusion to restore normal function and physiology. In addition, mtDNA damage activates DNA repair pathways such as BER to process the oxidative- or alkylating-agent-induced mtDNA damage and resolve some of the repair intermediates. Furthermore, unrepaired mtDNA base damage has an ability to deregulate the mtDNA replication dynamics leading to replication stress or blockage. mtDNA damage has been implicated in a variety of bacterial pathogens to drive inflammation and disease—for example, intracellular pathogenic bacteria such as *Salmonella typhimurium* induces typhoid-toxin-dependent mtDNA damage, promotes the release of mtDNA into the cytosol, and triggers the cGAS-STING pathway (Xu et al., 2022; Chen et al., 2024). *Mycobaterium abscessus* and *Mycobacterium tuberculosis* also cause mtDNA damage, leading to inflammation via inflammasome activation or cGAS-STING signaling (Wiens and Ernst, 2016; Kim et al., 2020). *H*. *pylori* infection potentially impacts the mtDNA integrity and transitory alteration of mitochondrial import translocases and a dramatic upregulation of POLG and TFAM. Spontaneous as well as chronic infection induces excessive accumulation of mtDNA damage which leads to the release of mtDNA into the cytoplasm and activates cGAS/STING-dependent type I interferon response or activate other additional signaling pathways to promote inflammation- and infection-associated pathogenicity. Future risk assessment of patients may look for the potential link between a mutation in the MTS sequence of BER genes and the biological consequence of insufficient mt BER repair factors. In the future, the clinical relevance and the mechanism underlying the altered mtDNA dynamics with or without *H. pylori* infection probably will provide a new insight for cancer risk assessments and therapeutic planning across different stages of gastric cancer.
